# Cardiac Magnetic Resonance Imaging with Myocardial Strain Assessment Correlates with Cardiopulmonary Exercise Testing in Patients with Pectus Excavatum

**DOI:** 10.3390/diagnostics14232758

**Published:** 2024-12-07

**Authors:** André Lollert, Tariq Abu-Tair, Tilman Emrich, Karl-Friedrich Kreitner, Alexander Sterlin, Christoph Kampmann, Gundula Staatz

**Affiliations:** 1 Department of Diagnostic and Interventional Radiology, Section of Pediatric Radiology, Medical Center of the Johannes Gutenberg-University, 55131 Mainz, Germany; gundula.staatz@unimedizin-mainz.de; 2 Center for Diseases in Childhood and Adolescence, Division of Pediatric Cardiology and Congenital Heart Diseases, Medical Center of the Johannes Gutenberg-University, 55131 Mainz, Germany; tariq.abu-tair@unimedizin-mainz.de (T.A.-T.); kampmann@uni-mainz.de (C.K.); 3 Department of Diagnostic and Interventional Radiology, Medical Center of the Johannes Gutenberg-University, 55131 Mainz, Germany; tilman.emrich@unimedizin-mainz.de (T.E.); karl-friedrich.kreitner@unimedizin-mainz.de (K.-F.K.); 4 Division of Cardiovascular Imaging, Department of Radiology and Radiological Science, Medical University of South Carolina, Charleston, SC 29425, USA; 5 German Centre for Cardiovascular Research, Partner Site Rhine-Main, 55131 Mainz, Germany; 6 Department of Pediatric Surgery, Medical Center of the Johannes Gutenberg-University, 55131 Mainz, Germany; alexander.sterlin@unimedizin-mainz.de

**Keywords:** pectus excavatum, magnetic resonance imaging, strain imaging, cardiopulmonary exercise testing

## Abstract

**Objectives**: To evaluate correlations between cardiac magnetic resonance imaging (cMRI) at rest including strain imaging and variables derived from quantitative cardiopulmonary exercise testing using a treadmill in patients with pectus excavatum. **Methods**: We retrospectively correlated the results of cMRI and cardiopulmonary exercise testing in 17 patients with pectus excavatum, in whom both examinations were performed during their pre-operative clinical evaluation. In addition to cardiac volumetry, we assessed the strain rates of both ventricles using a feature-tracking algorithm of a piece of commercially available post-processing software. **Results**: Right ventricular (RV) ejection fraction correlated negatively with heart rate at anaerobic threshold (rho = −0.543, *p* = 0.024). A positive correlation between radial strain rate at the RV base and percentage of predicted maximum heart rate (rho = 0.72, *p* = 0.001) was shown, with equivalent results for circumferential strain rate (rho = −0.64, *p* = 0.005). Radial strain rate at the RV base correlated in a strongly negative way with maximum oxygen uptake (rho = −0.8, *p* < 0.001), with a correspondingly positive correlation for circumferential strain rate (rho = 0.73, *p* = 0.001). **Conclusions**: Quantitative parameters derived from cMRI at rest, especially those acquired at the most severely compressed RV base, correlated with cardiopulmonary exercise testing variables. The compression of the RV base by the sternum might be partially compensated by an increased strain rate to induce higher heart frequencies during exercise. However, high strain rates were associated with a higher disease severity and a lower maximum oxygen uptake, indicating a limitation of this compensation mechanism.

## 1. Introduction

Pectus excavatum (PE), the most common congenital deformity of the anterior chest wall, is characterized by a depression of the sternum into the thoracic cavity. It occurs in approximately 1 of 400 live births with a male predominance (male-to-female ratio 4:1) [1]. The deformity can be quantified by measuring static morphometric parameters such as the Haller Index or Correction Index [2]. They reflect a measure of sternal depression in relation to outer thoracic diameters, and are ideally assessed by magnetic resonance imaging (MRI) due to the lack of ionizing radiation.

In patients with PE, sternal depression frequently leads to a compression of the atria (or atrioventricular groove) and right ventricle (RV) [3]. In addition, slight pericardial effusion is common. In some cases, the entire heart is dislocated to the left [4]. Several cardiac MRI studies have reported functional impairment, especially affecting the RV. Most of these studies assessed standard volumetric analyses. The main results consisted of a moderately reduced right ventricular ejection fraction in patients with PE [5,6,7].

Magnetic resonance strain imaging using a feature-tracking algorithm [8] based on standard steady-state free-precession sequences is a method that can further characterize and quantify myocardial contraction. Longitudinal strain is a measure for ventricular shortening from the base to the apex during the cardiac cycle. Circumferential strain quantifies myocardial fiber shortening along the circular perimeter. As both parameters quantify a shortening process, they are represented by negative numbers. In contrast, radial strain reflects ventricular thickening, and is therefore expressed in positive values [9].

RV strain alterations in patients with PE have been described, possibly being affected by the site of maximum compression [10,11,12]. One group also found subtle left ventricular dysfunction, represented by a reduced longitudinal strain [13]. However, the main methodological limitation of cardiac MRI is that it is performed at rest.

As patients with PE frequently suffer from limitations, especially during physical activities, exercise tests are performed to assess cardiopulmonary function [14]. In most studies, maximum oxygen uptake (VO_2max_) is one of the assessed endpoints [15]. Pre-therapeutic evaluation of impaired cardiac and pulmonary [16] function is relevant for treatment indications, as it can improve after surgery [15]. An increase in Haller and Correction Indices, indicating a more severe deformity, has been associated with worse parameters of cardiopulmonary function assessed by treadmill exercise testing [14]. However, there are no studies to evaluate correlations between these functional parameters and cardiac MRI. In addition, the clinical significance remains unclear, that is, whether both methods might be useful for the pre-therapeutic assessment, which is still not standardized across all centers. This also applies to the economic question of when to treat a patient surgically. Concerning this question, cardiac MRI is currently not recommended as a standard procedure by expert consensus [17]. Therefore, the objective of this study was to assess correlations between cardiopulmonary exercise testing and advanced cardiac MRI including strain imaging.

## 2. Materials and Methods

Approval by the local Independent Ethics Committee was not necessary according to local regulations due to the retrospective study design. The study population consisted of all patients with PE who underwent both cardiac MRI and treadmill exercise testing at our institution in 2015 and 2016. From 2017, no more patients could be included due to a change in the local pre-therapeutic evaluation procedures (cardiopulmonary exercise testing was omitted from the institutional standard of care).

### 2.1. MRI

All patients, or their legal guardians (if <18 years of age), provided written informed consent for the clinically indicated MRI examinations. All examinations were performed on a 3 Tesla scanner (Magnetom Skyra^®^, Siemens Healthineers, Erlangen, Germany).

Anatomic transverse T2-weighted half-Fourier acquisition single-shot turbo spin echo (HASTE) sequences (repetition time 500 ms, echo time 24 ms, slice thickness 5 mm, flip angle 130°, field of view 400 mm) were performed to measure the Haller Index and Correction Index in inspiration and expiration, as previously described [2]. The Haller Index is calculated by dividing the maximum latero-lateral width of the thorax skeleton through the distance between the vertebral spine and the sternum at the level of the deepest sternal depression. The Correction Index is a measure to relate the vertebro-sternal distance to the distance between the spine and the most anterior rib at the same level. Both indices increase in more severe deformities, and thresholds to indicate a PE deformity are 3.25 (Haller Index) and 10 (Correction Index), respectively.

In addition, we performed cardiac MRI, as described before [10]. We used standard steady-state free-precession (SSFP) sequences in the two- and four-chamber view (repetition time 3.27 ms, echo time 1.43 ms, flip angle 65°, slice thickness 6 mm, matrix 208 × 208, and field of view 340 mm). SSFP sequences in the short-axis view were conducted with slightly different imaging parameters (repetition time 3.32 ms, echo time 1.46 ms, flip angle 65°, slice thickness 8 mm, matrix 224 × 224, and field of view 340 mm). Parallel imaging was performed with the Generalized Autocalibrating Partially Parallel Acquisition (GRAPPA) algorithm with an acceleration factor of 3.

We performed standard cardiac volumetry to measure end-systolic/end-diastolic volumes, stroke volumes, cardiac indices, and ejection fractions for both ventricles. Subsequently, these parameters were normalized for body surface area. Biventricular myocardial strain analyses were performed using dedicated software (cvi^42^, version 5.11, Circle Cardiovascular Imaging, Calgary, AB, Canada). Peak systolic circumferential and radial strain rates were assessed using short-axis views at a basal, mid, and apical slice. Circumferential strain rate is a measure of myocardial shortening along the circular perimeter; radial strain describes the thickening myocardial deformation towards the center of the ventricles’ cavity per second. Peak systolic longitudinal strain rates, representing the rate of base–apex shortening, were evaluated using the four-chamber view. We used strain rates instead of strain for our further analyses, as they are less dependent on loading conditions [18].

### 2.2. Cardiopulmonary Exercise Testing

Cardiopulmonary exercise testing (CPET) was performed on an H/P/Cosmos treadmill using CareFusionMS-CPX 5.70 software (CareFusion, Höchberg, Germany) combined with GE-electrocardiogram Cardiosoft 6.7 (GE Healthcare, Chicago, IL, USA). We used an altered, improved treadmill protocol, as recommended by the German Society of Pediatric Cardiology [14,19]. Patients started the test at a speed of 2 km/h on a flat treadmill. We accelerated the speed by 0.5 km/h every 90 s; treadmill inclination was increased by 3% every 90 s up to a maximum of 21%. We stopped the test with a recovery phase on a flat treadmill with a speed of 2 km/h when the calculated target heart rate of 220 minus age in years (beats per minute) was reached or when the patient was completely exhausted. We assessed the following parameters: maximum oxygen uptake (VO_2max_), percentage of predicted oxygen uptake (VO_2max_%), oxygen uptake at anaerobic threshold (VO_2AT_), maximum oxygen pulse (O_2_-pulse_max_), percentage of predicted oxygen pulse (O_2_-pulse_max_%), oxygen pulse at anaerobic threshold (O_2_-pulse_AT_), maximum physical work capacity (Watt_max_), maximum heart rate (HR_max_), percentage of predicted heart rate (HR_max_%), and heart rate at anaerobic threshold (HR_AT_). Cardiopulmonary impairment was defined as a reduction in VO_2max_% of less than 85% of the predicted value.

### 2.3. Statistics

Statistical evaluations were performed with SPSS Statistics (Statistical Package for the Social Sciences, version 23.0; IBM, Armonk, NY, USA). We used the Mann–Whitney U test to assess gender differences in strain rates and CPET variables. Spearman’s rho was calculated to assess correlations between cardiac MRI and parameters derived from CPET. A *p*-value lower than 0.05 was considered significant. The analyses were exploratory; no adjustments for multiple testing were made.

## 3. Results

The final study cohort consisted of 17 patients (12 male, 5 female). The mean age was 15.5 years with a range from 10 to 25 years. The mean body mass index was 17.92 kg/m^2^ (standard deviation [SD]: 2.43 kg/m^2^). Nine patients had a symmetric deformity, eight patients an asymmetric deformity. The mean Haller Index was 4.18 (SD: 0.82, range 3.2–6.53) in inspiration and 4.95 (SD: 1.21, range 3.69–8.23) in expiration. The mean Correction Index was 26.1 (SD: 12.3, range 7.1–52.9) in inspiration and 28.3 (SD: 14.5, range 7.7–56.9) in expiration. Both the Haller Index (inspiration: *p* = 0.383; expiration: *p* = 0.279) and the Correction Index (inspiration: *p* = 0.195; expiration: *p* = 0.16) were not significantly different in males versus females.

### 3.1. Cardiac MRI

Results of standard cardiac volumetry are demonstrated in Table 1.

**Figure 1:**
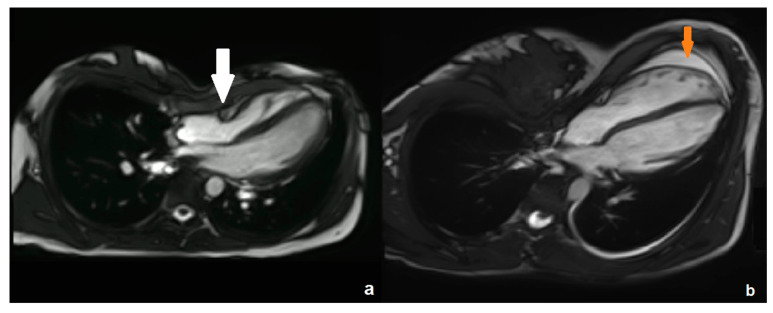
An example of a steady-state free-precession cardiac MRI sequence demonstrates the most frequent type of compression of the RV base by the sternum ((**a**), white arrow). In a second example (**b**), a shift of the entire heart to the left is depicted in addition to a pericardial effusion (orange arrow).

All left ventricular (LV) parameters were within the normal range. A total of 5 of 17 patients (29%) had a reduced RV ejection fraction (<45%). In four patients, the normalized end-diastolic RV volume was elevated (>100 mL/m^2^). Figure 1 exemplarily demonstrates two of the MRI examinations, with typical compression of the RV base by the sternum (a) and a shift of the entire heart to the left (b).

The results of the strain analyses are shown in Table 2.

We demonstrated an increase in both circumferential and radial strain rates from both ventricles’ bases to their apices. LV global radial strain rate was significantly lower in males than in females (mean ± standard deviation 2.5 ± 0.43/s versus 3.1 ± 0.37/s, *p* = 0.019); all other strain parameters did not significantly differ by gender.

### 3.2. Cardiopulmonary Exercise Testing

The CPET results are summarized in Table 3.

Cardiopulmonary impairment, defined as a reduction in VO_2max_% of less than 85% of the predicted value, was demonstrated in 3/17 patients (18%). Despite a significantly lower VO_2max_% in males versus females (mean ± standard deviation 92 ± 13.4% versus 113 ± 13.9%, *p* = 0.027), no significant gender differences were found for the remaining CPET parameters.

### 3.3. Correlations Between Cardiac MRI and CPET Data

**Figure 2:**
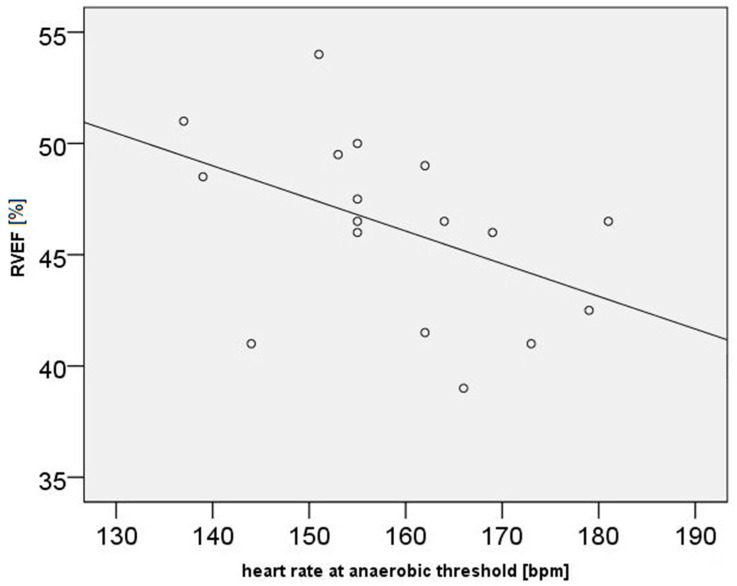
A significant negative correlation between RVEF and HR_AT_ is shown.

LV ejection fraction correlated negatively with VO_2max_ (rho = −0.63, *p* = 0.007), O_2_-pulse_max_% (rho = −0.52, *p* = 0.034), and Watt_max_ (rho = −0.51, *p* = 0.035). RV ejection fraction correlated negatively with HR_AT_ (rho = −0.54, *p* = 0.024, Figure 2).

Correlation coefficients between cardiac volumetry and CPET parameters are shown in Supplementary Table S1.

LV global radial strain rate correlated negatively with VO_2max_ (rho = −0.69, *p* = 0.002), with a corresponding positive correlation for mid-ventricular circumferential strain rate (rho = 0.57, *p* = 0.017). LV apical radial strain rate correlated negatively with HR_AT_ (rho = −0.54, *p* = 0.025), with a corresponding positive correlation for circumferential strain rate (rho = 0.49, *p* = 0.048). All other LV strain rates did not correlate with CPET data.

**Figure 3:**
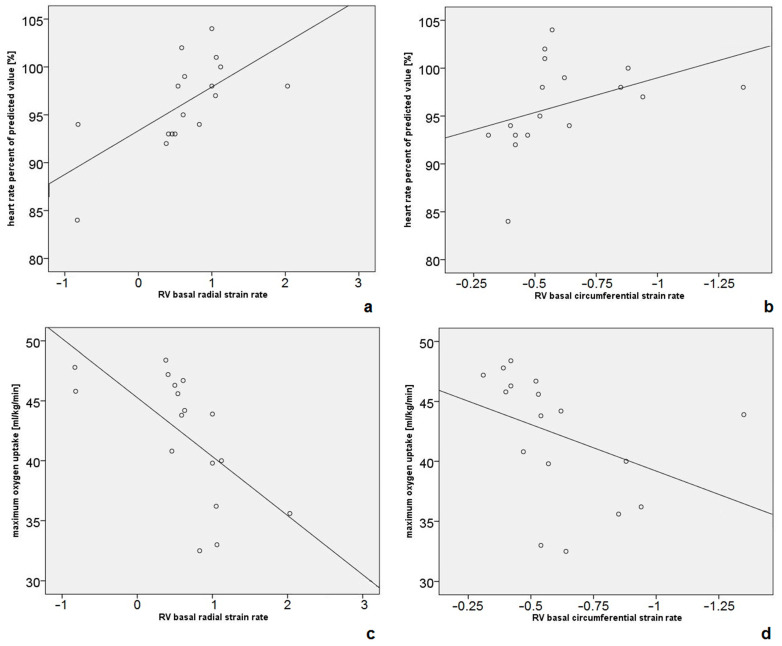
RV basal radial and circumferential strain rates correlate in a significantly positive way with HR_max_% (**a**,**b**) and in a negative way with VO_2max_ (**c**,**d**). For a better visualization, circumferential strain rates are depicted descending from 0 on the x axis, so that higher strain rates are always on the right side of the diagram despite being measured by negative values.

We did not find significant correlations between RV longitudinal strain rate and any of the CPET parameters. Correlations between RV radial and circumferential strain rates and CPET data are shown in Supplementary Table S2. Positive correlations between RV basal radial strain rates and HR_max_ (rho = 0.71, *p* = 0.002), as well as HR_max_% (rho = 0.72, *p* = 0.001, Figure 3a), were demonstrated. Circumferential strain rates corresponded to the aforementioned values (HR_max_: rho = −0.53, *p* = 0.028; HR_max_%: rho = 0.72, *p* = 0.001, Figure 3b). In addition, RV basal radial strain rate correlated negatively with VO_2max_ (rho = −0.8, *p* < 0.001, Figure 3c), with a corresponding positive correlation for circumferential strain rate (rho = 0.73, *p* = 0.001, Figure 3d).

An additional analysis demonstrated a positive correlation between RV basal radial strain rate and the Haller Index (rho = 0.53, *p* = 0.03), as well as the Correction Index (rho = 0.638, *p* = 0.006) in expiration.

## 4. Discussion

In this study, correlations between cardiac MRI data obtained at rest and CPET data obtained during physical activity were demonstrated. In particular, the RVEF correlated negatively with the heart rate at anaerobic threshold. Strain rates at the usually most compressed heart compartment, the RV base [10,20], correlated in a significantly positive way with maximum heart rates and their percentage of the predicted value. In addition, a significantly negative correlation with the maximum oxygen uptake was shown.

For the left ventricle, the EF and global radial strain rate, as another measure of contractility [9], were associated with a lower VO_2max_, O_2_-pulse_max_%, and Watt_max_. As the LV is usually not compressed by the thoracic wall deformity, this is most probably explained by the general physical training condition of the patients [21]. However, one group of authors found signs of a subtle LV dysfunction in patients with PE, represented by a reduced global longitudinal strain [13]. This might contribute to physical impairment, as expressed by the aforementioned CPET parameters. Still, it remains unclear why LV longitudinal strain rates did not correlate with CPET data in our study. One biomechanical explanation might be the fact that, in PE, the sternum mainly compresses the interventricular septum perpendicular to the long-axis direction, while the lateral ventricular wall can contract freely.

For the right ventricle, which is directly compressed by the sternum in patients with PE, this anatomic obstruction must be taken into account to explain the aforementioned correlations between cardiac MRI parameters obtained at rest and dynamic CPET data. Previous studies demonstrated abnormally decreased RVEFs in some patients with PE [3,7,10]. Especially in severe cases, exercise tolerance is impaired [22]. In general, a reduced exercise capacity can be expected if the RVEF is low. For example, this was shown in a cohort of patients with repaired tetralogy of Fallot, who were of similar ages compared with our cohort [23]. However, as patients with PE usually do not present with an additional primary heart disease, their heart might be capable of compensating for the anatomic compression. We demonstrated a negative correlation between RVEF at rest and HR_AT_, that is, HR_AT_ was higher in patients with a low RVEF. As an increased HR_AT_ usually indicates an enhanced cardiac output [24], this might contribute to compensating for the lower RV stroke volume (due to external compression). This hypothesis is supported by the fact that, in the study of Abu-Tair et al. [14], HR_AT_ was the first CPET parameter to react/increase in patients with a PE deformity with a pathological Haller Index of >3.25.

Myocardial strain measurements allow for a more subtle assessment of ventricular contractility and function compared with volumetry alone [9]. Thus, they might serve as markers of additional compensatory mechanisms to maintain RV function (which is still normal in the majority of patients with PE). In our study, an increased strain rate at the RV base correlated with a higher HR_max_ and HR_max_%. This might reflect a compensatory mechanism to increase regional contractility and enhance RV output by frequency. This hypothesis is supported by the positive correlation between RV basal radial strain rate and the Haller Index and Correction Index, which means that strain rates were higher in more severe deformities. Still, when regarding the most widely used surrogate marker of exercise capacity (VO_2max_), we demonstrated a negative correlation with RV basal strain rates. As VO_2max_ is a marker which deteriorates especially in severe forms of PE [4,14], this might indicate that the aforementioned potential compensatory mechanisms are not sufficient to maintain full exercise capacity in severely affected individuals.

To date, several studies have assessed and confirmed that heart function is influenced by the presence of a PE chest wall deformity [25]. However, little is known about the underlying pathophysiology [26]. One study even demonstrated a gender difference, with females showing better cardiopulmonary function and exercise tolerance than males even in cases of more severe anatomic compression [27]. This was partly confirmed by our results as VO_2max_% was higher in females than in males, whereas the Haller Index and Correction Index did not significantly differ by gender. To our knowledge, there are no other studies comparing static cardiac MRI with CPET data, making it difficult to compare our results with the literature. Therefore, there is a need for research on how the PE deformity affects heart physiology under workload. Probably, treadmill exercise cardiac MRI [28] might also be used to further characterize the deformity. Still, despite having demonstrated inter-method correlations and a potential pathophysiology mechanism, our results can only be seen as a first step towards standardization of the clinical evaluation of patients with PE. Further research is needed to assess whether advanced methods such as cardiac MRI or CPET are necessary for all of these patients.

### Limitations

Our study has several limitations. First, due to the retrospective study design and omission of CPET evaluations from the clinical standard evaluation, the sample size is small. However, we were able to show that there are possible connections between MRI data obtained at rest and dynamic CPET data obtained during exercise, which should be confirmed in larger studies. In addition, a matching analysis study to compare the results of patients with PE with healthy probands would be helpful to appraise the data more precisely. Second, a causal correlation between the assessed markers cannot be derived from the data. Therefore, these data have to be interpreted with caution, especially concerning potential pathophysiologic processes (i.e., potential compensatory mechanisms). Third, we only assessed pre-operative data. As improvements in cardiopulmonary function are expected after surgery [29,30], it would be helpful to see if correlations between MRI and CPET data change after surgical repair of the deformity. Last, a methodological limitation of cardiac strain MRI is the fact that right ventricular circumferential and radial strain assessments, especially at the base, have a lower reproducibility compared with the left ventricle due to the thinner myocardium [9].

## 5. Conclusions

In conclusion, quantitative parameters derived from cardiac MRI at rest, especially those acquired at the most severely compressed RV base, correlated with CPET variables. The compression of the RV base by the sternum might be partially compensated by an increased strain rate to induce higher heart frequencies during exercise. However, a limitation of compensatory possibilities in cases of severe compression is reflected by a lower maximum oxygen uptake, even when strain rates are high. These findings are of clinical relevance as they further characterize the influence of the deformity on cardiopulmonary function both at rest and during exercise. Further research is needed to assess if strain measurements or a combination with CPET data might be used to stratify patients for potential post-therapeutic improvement of cardiopulmonary function in the future.

## Figures and Tables

**Table 1:** Parameters derived from standard cardiac MRI volumetry.

Parameter	Mean	Standard Deviation	Minimum	Maximum
Heart rate [beats per minute]	74	10	61	95
Left ventricle				
- normalized end-diastolic volume [mL/m^2^]	78.5	9.8	62	92
- normalized end-systolic volume [mL/m^2^]	28.2	5	20	36
- normalized stroke volume [mL/m^2^]	50.2	6	42	61
- cardiac index [L/min/m^2^]	3.83	0.63	3.1	5.6
- ejection fraction [%]	64	3.4	58	70
Right ventricle				
- normalized end-diastolic volume [mL/m^2^]	94.6	11.6	72	115
- normalized end-systolic volume [mL/m^2^]	50.8	8.1	36	67
- normalized stroke volume [mL/m^2^]	42.8	5.7	34	54
- cardiac index [L/min/m^2^]	3.26	0.37	2.8	4.5
- ejection fraction [%]	46	4.1	39	54

**Table 2:** Parameters derived from cardiac MRI strain imaging (in the circumferential orientation, a higher strain rate is characterized by more negative values).

Parameter	Mean	Standard Deviation	Minimum	Maximum
Left ventricle				
Circumferential strain rate [s^−1^]				
- global	−1.43	0.2	−1.83	−1.09
- basal	−1.23	0.27	−1.76	−0.74
- mid-ventricular	−1.33	0.26	−2.02	−0.95
- apical	−2.08	0.47	−3.06	−1.02
Radial strain rate [s^−1^]				
- global	2.68	0.49	2.05	3.61
- basal	2.3	0.8	1.39	3.99
- mid-ventricular	2.67	0.56	1.82	3.8
- apical	3.63	0.78	2.36	5.68
Global longitudinal strain rate [s^−1^]	−1.09	0.33	−1.88	−0.57
Right ventricle				
Circumferential strain rate [s^−1^]				
- global	−0.72	0.2	−1.07	−0.39
- basal	−0.61	0.26	−1.35	−0.31
- mid-ventricular	−0.71	0.21	−1.21	−0.29
- apical	−1.03	0.24	−1.56	−0.68
Radial strain rate [s^−1^]				
- global	1.06	0.32	0.54	1.47
- basal	0.62	0.67	0.83	2.03
- mid-ventricular	0.95	0.32	0.32	1.7
- apical	1.55	0.4	0.92	2.28
Global longitudinal strain rate [s^−1^]	−1.15	0.24	−1.51	−0.82

**Table 3:** Parameters derived from cardiopulmonary exercise testing.

Parameter	Mean	Standard Deviation	Minimum	Maximum
Maximum oxygen uptake (VO_2max_, mL/kg × min)	42.2	5.2	32.5	48.4
Percentage of predicted oxygen uptake (VO_2max_%)	98.3	16.2	69.6	127.7
Oxygen uptake at anaerobic threshold (VO_2AT_, mL/kg × min)	26.3	4.4	17.7	34.7
Maximum oxygen pulse (O_2_-pulse_max_, mL/beat)	11.1	2.6	7.2	15.9
Percentage of predicted oxygen pulse (O_2_-pulse_max_%)	87.7	15.3	53.6	110.3
Oxygen pulse at anaerobic threshold (O_2_-pulse_AT_, mL/beat)	8.5	1.9	5.3	12
Maximum physical work capacity (Watt_max_, Watt/kg)	4.6	0.56	3.5	5.5
Maximum heart rate (HR_max_, beats/min)	197	8	173	206
Percentage of predicted heart rate (HR_max_%)	96.2	4.7	84	104
Heart rate at anaerobic threshold (HR_AT_, beats/min)	159	12.6	137	181

## Data Availability

A.L. and G.S. had full access to all the data in the study and take responsibility for the integrity of the data and the accuracy of the data analysis. The data presented in this study are available on request from the corresponding author. The data are not publicly available due to privacy (medical data).

## Supplementary Materials

The following supporting information can be downloaded at https://www.mdpi.com/article/10.3390/diagnostics14232758/s1, Table S1: Correlation coefficients between cardiac MRI volumetric parameters and CPET data; Table S2: Correlation coefficients between right ventricular radial and circumferential strain rates and CPET data.
