# Supplementary material for: Cardiac Magnetic Resonance Imaging with Myocardial Strain Assessment Correlates with Cardiopulmonary Exercise Testing in Patients with Pectus Excavatum
Source: Diagnostics (Basel). 2024 Dec 7;14(23):2758. doi: 10.3390/diagnostics14232758 (PMC11640324; doi:10.3390/diagnostics14232758)
Supplement: Supplementary file 1 [file diagnostics-14-02758-s001.zip › SupplTable1.pdf]

	VO_{2max}	$VO_{2max\%}$	VO_{2AT}	O_2-pulse$_{max}$	O_2-pulse$_{max\%}$	O_2-pulse$_{AT}$	Watt$_{max}$	HR$_{max}$	HR$_{max\%}$	HR$_{AT}$
LV-EDVn<br>P	**0.56**<br>**0.019**	0.06<br>0.812	0.21<br>0.416	0.41<br>0.098	-0.35<br>0.176	0.26<br>0.313	0.28<br>0.278	-0.04<br>0.871	-0.19<br>0.462	0.12<br>0.635
LV-ESVn<br>P	**0.71**<br>**0.001**	0.05<br>0.844	0.3<br>0.248	**0.53**<br>**0.027**	-0.32<br>0.206	0.38<br>0.132	0.42<br>0.094	-0.12<br>0.636	-0.29<br>0.263	0.09<br>0.72
LV-SVn<br>P	0.34<br>0.182	0.05<br>0.837	0.03<br>0.899	0.2<br>0.447	-0.4<br>0.116	0.04<br>0.892	0.06<br>0.809	0.09<br>0.734	-0.09<br>0.727	0.15<br>0.555
LV-CI<br>P	-0.13<br>0.626	-0.15<br>0.579	-0.1<br>0.695	0.09<br>0.718	-0.21<br>0.425	0.06<br>0.833	-0.02<br>0.946	0.24<br>0.358	0.13<br>0.634	0.14<br>0.603
LVEF<br>P	**-0.63**<br>**0.007**	-0.03<br>0.914	-0.42<br>0.097	**-0.52**<br>**0.034**	0<br>1	-0.48<br>0.052	**-0.51**<br>**0.035**	0.19<br>0.468	0.22<br>0.398	-0.07<br>0.804
RV-EDVn<br>P	0.34<br>0.184	-0.03<br>0.922	0.3<br>0.243	0.31<br>0.226	-0.34<br>0.176	0.18<br>0.484	0.29<br>0.259	0.2<br>0.453	0.11<br>0.678	**0.57**<br>**0.017**
RV-ESVn<br>P	0.2<br>0.45	-0.06<br>0.826	0.18<br>0.496	0.2<br>0.45	-0.4<br>0.113	0.05<br>0.852	0.24<br>0.356	0.31<br>0.233	0.19<br>0.47	**0.68**<br>**0.003**
RV-SVn<br>P	**0.53**<br>**0.029**	0.15<br>0.576	0.29<br>0.251	0.34<br>0.188	-0.18<br>0.482	0.22<br>0.395	0.24<br>0.348	-0.15<br>0.576	-0.2<br>0.446	0.07<br>0.795
RV-CI<br>P	0.08<br>0.753	-0.02<br>0.929	0.18<br>0.489	0.24<br>0.357	-0.05<br>0.837	0.22<br>0.388	0.02<br>0.949	0.24<br>0.353	0.18<br>0.484	0.16<br>0.536
RVEF<br>P	0.18<br>0.493	0.07<br>0.8	0.12<br>0.645	0.2<br>0.452	0.34<br>0.181	0.28<br>0.28	-0.07<br>0.785	-0.38<br>0.129	-0.3<br>0.238	**-0.54**<br>**0.024**

Supplementary Table S1 – Correlation coefficients (Spearman's rho) between cardiac MRI volumetric data and cardiopulmonary exercise testing parameters. Significant correlations are marked in bold letters.
