# Supplementary material for: Cardiac Magnetic Resonance Imaging with Myocardial Strain Assessment Correlates with Cardiopulmonary Exercise Testing in Patients with Pectus Excavatum
Source: Diagnostics (Basel). 2024 Dec 7;14(23):2758. doi: 10.3390/diagnostics14232758 (PMC11640324; doi:10.3390/diagnostics14232758)
Supplement: Supplementary file 1 [file diagnostics-14-02758-s001.zip › SupplTable2.pdf]

	VO_{2max}	$VO_{2max\%}$	VO_{2AT}	O_2-pulse$_{max}$	O_2-pulse$_{max\%}$	O_2-pulse$_{AT}$	Watt$_{max}$	HR_{max}	$HR_{max\%}$	HR_{AT}
RV radial SR										
global	-0.46	-0.29	-0.35	0.3	0.24	0.3	0.22	0.18	0.33	0.04
P	0.061	0.266	0.172	0.245	0.355	0.245	0.404	0.494	0.197	0.884
basal	**-0.8**	-0.37	-0.46	-0.26	0.12	-0.24	-0.36	**0.71**	**0.72**	0.28
P	**<0.001**	0.142	0.065	0.314	0.653	0.347	0.155	**0.002**	**0.001**	0.279
mid	-0.42	0.02	-0.39	0.09	0.19	0.12	0.22	0.04	0.19	-0.06
P	0.091	0.948	0.128	0.718	0.468	0.64	0.39	0.879	0.473	0.81
apical	-0.06	-0.2	0.03	**0.54**	0.33	**0.56**	0.4	0.06	0.19	-0.01
P	0.808	0.445	0.896	**0.024**	0.195	**0.019**	0.117	0.829	0.466	0.97
RV circumf. SR										
global	**0.56**	0.12	0.23	-0.12	-0.37	-0.17	-0.07	-0.42	**-0.54**	-0.18
P	**0.02**	0.66	0.373	0.639	0.147	0.519	0.782	0.094	**0.027**	0.495
basal	**0.73**	0.3	0.34	0.14	-0.34	0.09	0.17	**-0.53**	**-0.64**	-0.23
P	**0.001**	0.239	0.18	0.599	0.187	0.731	0.505	**0.028**	**0.005**	0.375
mid	**0.61**	0.17	**0.59**	0.18	0.01	0.16	0.12	-0.18	-0.25	0.14
P	**0.01**	0.504	**0.012**	0.492	0.974	0.54	0.65	0.491	0.339	0.587
apical	-0.05	-0.1	-0.13	-0.38	-0.39	-0.41	-0.31	-0.04	-0.15	0.05
P	0.841	0.69	0.62	0.136	0.125	0.104	0.225	0.892	0.564	0.852

Supplementary Table S2 – Correlation coefficients (Spearman's rho) between right ventricular (RV) strain rates (SR) derived from cardiac MRI and cardiopulmonary exercise testing parameters. Significant correlations are marked in bold letters.
